# Histological and Radiological Features of a Four-Phase Injectable Synthetic Bone Graft in Guided Bone Regeneration: A Case Report

**DOI:** 10.3390/ijerph18010206

**Published:** 2020-12-29

**Authors:** Marija Čandrlić, Željka Perić Kačarević, Zrinka Ivanišević, Matej Tomas, Aleksandar Včev, Dario Faj, Marko Matijević

**Affiliations:** 1 Department of Dental Medicine, Faculty of Dental Medicine and Health Osijek, J.J. Strossmayer University of Osijek, 31 000 Osijek, Croatia; marija.candrlic@fdmz.hr (M.Č.); zrinkaivan@gmail.com (Z.I.); matej.tomas@fdmz.hr (M.T.); 2 Faculty of Medicine Osijek, J.J. Strossmayer University of Osijek, 31 000 Osijek, Croatia; 3 Department of Anatomy, Histology, Embriology, Pathology Anatomy and Pathology Histology, Faculty of Dental Medicine and Health Osijek, J.J. Strossmayer University of Osijek, 31 000 Osijek, Croatia; zpkacarevic@fdmz.hr; 4 Department of Pathophysiology, Physiology and Immunology, Faculty of Dental Medicine and Health Osijek, J.J. Strossmayer University of Osijek, 31 000 Osijek, Croatia; avcev@fdmz.hr; 5 Department of Biophysics and Radiology, Faculty of Dental Medicine and Health Osijek, J.J. Strossmayer University of Osijek, 31 000 Osijek, Croatia; dario.faj@fdmz.hr

**Keywords:** injectable synthetic bone graft, guided bone regeneration, tissue engineering, biomaterial

## Abstract

Background and objective: Injectable synthetic bone grafts (ISBG) are widely used biomaterials for regeneration purposes. The aim of this case report was to examine the efficacy of ISBG in the management of buccal fenestration in the case of a 25-year-old female. Case report: After a traumatic tooth extraction, the defect was filled with ISBG and covered with a resorbable membrane. The ISBG showed easy handling and the patient had no complications during healing. Six months after augmentation, a bone biopsy was taken during implant bed preparation. The histological results showed good integration of ISBG into the newly formed bone and no signs of tissue inflammation. Additionally, a CBCT (cone beam computed tomography) analysis was performed to support the histological results. Conclusion: The use of the examined ISBG led to successful treatment of the buccal fenestration defect.

## 1. Introduction

Failed endodontic treatments can often lead to teeth loss and implant-supported prosthetics is the most promising solution for treating such cases [1,2,3]. However, that is quite challenging due to frequent bone loss caused by periapical lesions [4]. More specifically, significant changes in alveolar ridge dimensions occur mostly during the first year following extraction [5,6,7]. Therefore, an augmentation procedure before implant placement is recommended in order to ensure adequate alveolar bone volume [8,9,10,11,12].

Guided bone regeneration (GBR) is reliable and one of the most frequently used surgical techniques in implant dentistry [13,14,15]. Various bone grafting substitutes (allograft, xenograft, synthetic) are being used in combination with resorbable and non-resorbable barrier membranes in order to perform successful GBR [16,17,18]. More specifically, the synthetic graft substitutes support regenerative mechanisms that rely on both new bone formation in porosity and on actual remodeling of the graft into new bone [17]. Here the increase in graft porosity during remodeling is a parallel mechanism to decreased architectural structure provided by the initial grafting volume. Therefore, it is essential to properly balance the hydroxyapatite (HA, low biodegradation rate) and ß-tricalcium phosphate (ß-TCP, high biodegradation rate) ratio when creating biphasic synthetic substitutes. It is well known that in patients, the biphasic (60% HA and 40% ß-TCP) perform better than monophasic (ß-TCP) synthetic substitutes due to higher volume stability and homogeneity [19]. That is because ß-TCP lacks volume stability during remodeling and the presence of slow-resorbing HA imparts slower graft changes. The ideal homogenous biphasic composition seems to be 60% HA and 40% ß-TCP because it mirrors controlled resorption behavior and results in initial particle integration followed by complete resorption [20,21,22,23]. Here the fast resorbing ß-TCP continues to increase the material porosity that allows cell ingrowth, while HA provides volume stability for extended time periods.

Even though the addition of a third synthetic phase (HA nanoparticles in a water-based gel) seems to promote higher volumetric bone loss after three years, it still enables complete recovery of the bone defects and excellent volume stability over time [24,25]. This injectable synthetic bone graft (ISBG) (60% HA/40% ß-TCP granules and HA nanoparticles within water-based gel) can also build a barrier-like structure that is able to control soft tissue infiltration into the implanted bed [26]. For that reason, we assessed the histological outcome of GBR with such ISBG in patients having extensive chronic apical periodontitis that caused buccal fenestration. We aimed to achieve optimal implant stability and aesthetical results since delayed implantation was expected to ensure sufficient healing time of inflamed and missing bone. Therefore, tooth extraction was followed by augmentation with ISBG that was covered by native collagen membrane. Additionally, a CBCT (cone beam computed tomography) analysis was performed to support the histological results.

## 2. Case Report

**Figure 1:**
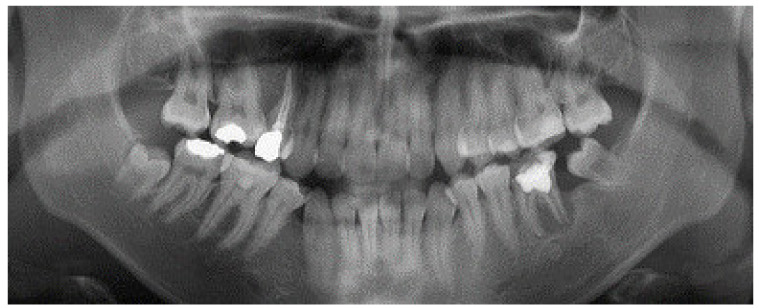
The radiographic evaluation showed severe bone loss in the distal root area of the hopeless tooth #19.

Approval for histological and radiological evaluation was obtained from the Ethics Committee of the Community Healthcare Centre Osijek (No. 03-1365-1/18), and a consent form was signed by the patient. A 25-year-old female with earlier endodontic treatment in the lower left quadrant, and neglected oral health in the past three years, was admitted to the Department of Oral surgery at Community Healthcare Center in Osijek. The patient had persistent pain with discomfort in the left maxillary and mandibular quadrants. There was no history of some specific illness, allergic diseases and unhealthy habits such as smoking or alcohol abuse. The examination showed several restoration treatments and the presence of supragingival plaque. Teeth #14 and #19 (ADA Dental Terminology 2011–2012) had old restorations and were sensitive during vertical and horizontal percussion. Periodontal probing of tooth #19 revealed a deep pocket on the distal root area. Orthopantomogram showed tooth #19 to have severe periapical bone destruction in the distal root region. Radiographic measurement revealed the vertical bone loss of 8.4 mm and horizontal bone loss of 9.8 mm. Tooth #14 had chronic apical periodontitis (Figure 1).

The oral surgeon suggested endodontic treatment on tooth #14. The patient underwent root canal treatment on tooth #19; however, during root canal cleaning, obstruction was detected in one of the mesial root canals and the root canal could not be properly sealed. In addition, the patient had persistent pain and developed a periapical abscess between visits during treatment. Accordingly, tooth #19 could not be properly treated and tooth extraction was the only option [27,28,29]. Therefore, a two-step implant treatment plan for tooth #19 was presented before obtaining the patient’s approval.

**Figure 2:**
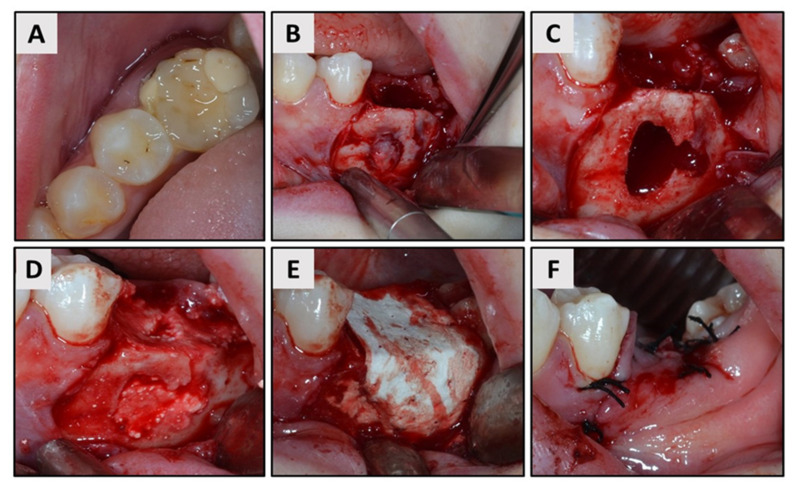
(**A**) Occlusal aspect of hopeless tooth #19. (**B**,**C**) Exposure of buccal fenestration after flap elevation and curettage of the infected tissue. (**D**) Application of injectable synthetic bone graft (ISBG) into the extraction socket. (**E**,**F**) Coverage of the defect with resorbable membrane and final wound closure.

First single dose of oral antibiotics (Klavocin 875 mg + 125 mg, Pliva, Zagreb, Croatia) was administered 1 h before surgery. Then local anesthesia (Ubistesin Forte 40 mg/mL + 0.01 mg/mL, 3M Deutschland GmbH, Seefeld, Germany) and mouth rinsing with chlorhexidine (Parodontax 0.2%, Brentford, London, UK) was applied before mucoperiosteal flap elevation to expose the extraction site. After a traumatic tooth extraction, buccal fenestration was observed and measured using a standard surgical caliper (straight Castroviejo caliper, Hu-Friedy, Chicago, IL, USA). The measurement revealed a defect size of 20.2 mm at the highest point of the defect and 21.3 mm at the widest point. Following detailed curettage of the infected tissue, the defect was filled with ISBG (maxresorb inject, botiss biomaterials GmbH, Berlin, Germany) and was covered by native collagen membrane (collprotect, botiss biomaterials GmbH, Berlin, Germany). Single 5/0 sutures were used for primary wound closure (Figure 2A–F).

Finally, the patient received detailed instructions on postoperative oral hygiene and oral antibiotic therapy, a combination of amoxicillin and clavulanic acid (Klavocin 875 mg + 125 mg, Pliva, Zagreb, Croatia), prescribed twice daily for 7 d to minimize the risk of infection. Ten days postoperatively the sutures were removed and CBCT was taken. Weekly follow-ups during the first month and monthly check-ups during the next five months were scheduled. Final CBCT examination was done just before the implant placement. The CBCT scans were done using a Scanora 3D (Soredex, Tuusula, Finland). The patient’s head was fixed in a standardized position during both scans. Correct positioning was verified using the scout preview images and imaging was performed using a standardized CBCT scan protocol. Then the 3D and axial images were reconstructed and saved in a DICOM format. The software used to analyze the DICOM data was OnDemand 3DApp version 1.0 (CyberMed International, Seoul, Korea). It was used to obtain a mean grey level using the region of interest (ROI) tool. The 1.7 mm × 3.6 mm rectangular ROI was located on the cross-sectional plane images at the site of augmentation. It is impossible to eliminate the factor of human error in selection of the ROI, so the ROI was taken 5 times and the average value of grey levels and standard deviations were taken. The images of each CBCT scan were captured using the image capture function in the OnDemand 3DApp software and exported into a Microsoft Word document for record keeping. However, the displayed grey levels in CBCT systems are not reliable and do not allow for the assessment of bone quality as it can be performed with HU (Hounsfield unit) in medical CT (computed tomography) [30]. In medical CT, HU provide a standard scheme for scaling the reconstructed attenuation coefficients. Though the manufacturers of dental CBCT systems do not use a standard system for scaling the grey levels representing the reconstructed values, it is possible to relate grey levels to the HU using simple method [31]. Since the patient in this case report was always imaged in the same conditions with the same CBCT scanning protocol, comparison of grey levels between images gave impressions of bone regeneration.

During implant bed preparation, a bone biopsy was taken using trephine bur (2.5 mm internal diameter; Ustomed instrumente, Tuttlingen, Germany) and then fixed in 4% formaldehyde solution. After two weeks of fixation in formaldehyde solution, the biopsy specimen was decalcified by solution (Solvagreen, Carl Roth, Karlsruhe, Austria). The next step was putting the specimen in a tissue processor (SLEE MTP, Mainz, Germany) and embedding in paraffin wax (SLEE MPS/P, Mainz, Germany). Paraffin blocks with biopsy were cut by microtome to 5 μm. These specimens were then stained with hematoxylin-eosin and Movat pentachrome. The stained sections were examined and recorded under a light microscope (Leica DMRB, Leica Microsystems GmbH, Wetzlar, Germany) connected to a video camera (Axio Imager M2, Zeiss, Oberkochen, Germany). The histomorphometric examination was done through digital image evaluation of photomicrographs stained by hematoxylin-eosin under 10× objective magnification. Histomorphometric image analysis was performed using ImageJ software (NIH, https://imagej.nih.gov/ij/). ImageJ was used to define the rectangular ROI window in three sections of the bone sample, separated in the central part by 50 μm. For each ROI, measurements of total area, bone tissue area, residual ISBG area and soft tissue area were taken and then transferred to a Microsoft Excel spreadsheet. Finally, the mean values of the above parameters were converted into volume percentages of mineralized tissue, ISBG and soft tissue.

## 3. Results

**Figure 3:**
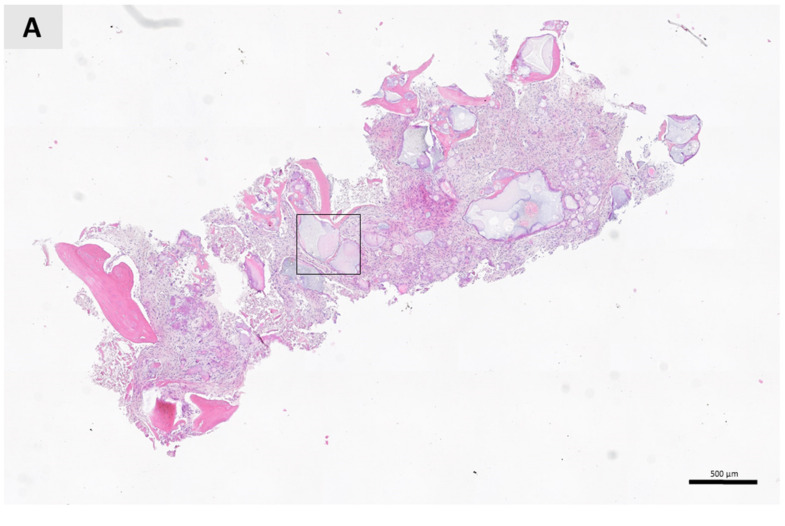

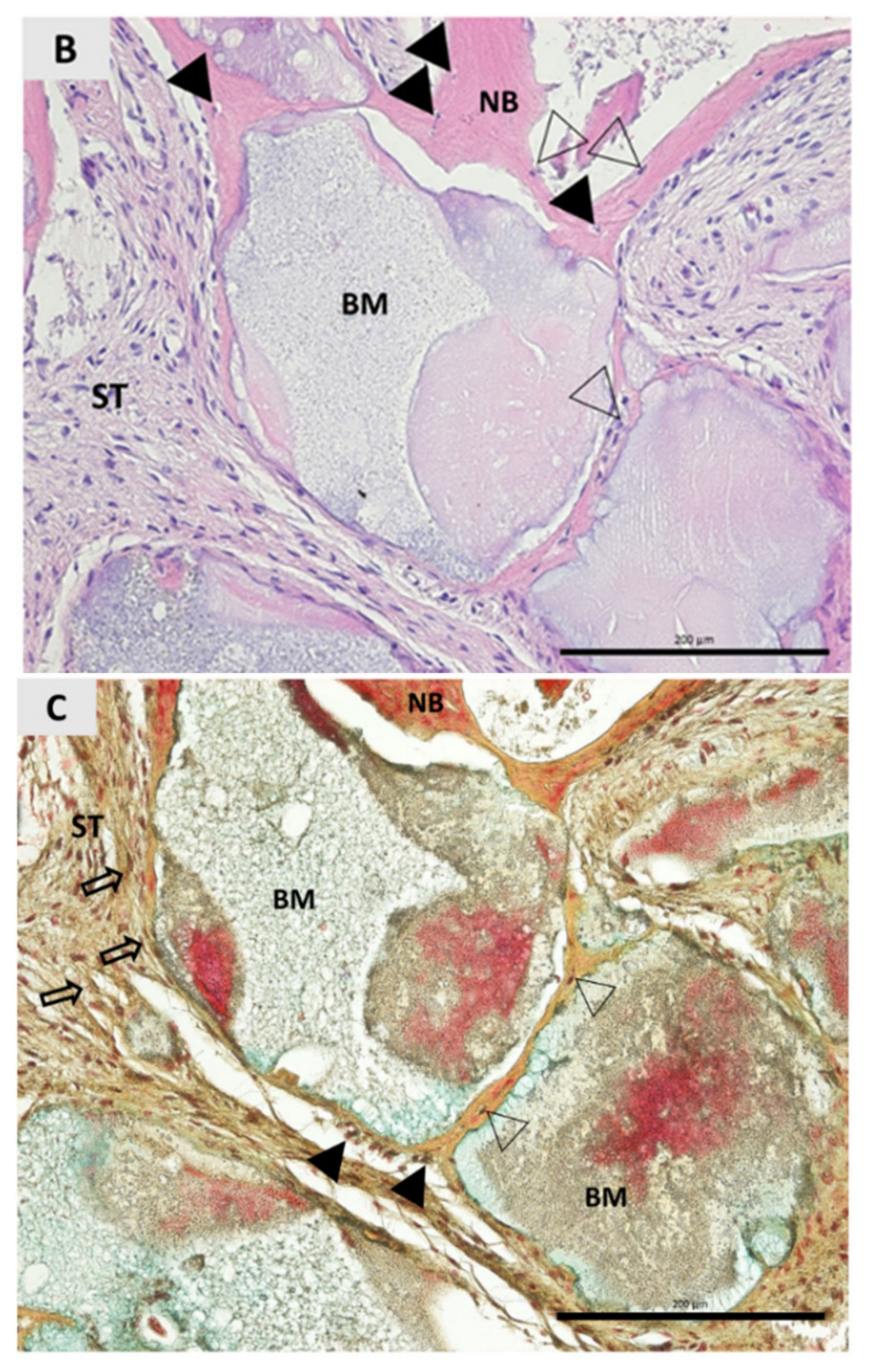
Histological examination of the bone biopsy taken six months post-implantation. (**A**) Longitudinal section of sample harvested from the augmented area. ISBG granules are completely consolidated with newly formed bone starting at the periphery border between the ISBG and pre-existing defect. The area of interest is marked by a square and shown in higher magnification (hematoxylin and eosin staining; magnification 10×). (**B**) Closer look at the area of interest. The residual ISBG is in close contact with a newly formed bone. There is no sign of inflammatory tissue response. Osteocytes are trapped into bone tissue, while active osteoblasts can be detected at the peripheral regions of residual ISBG and newly formed bone (hematoxylin and eosin staining; magnification 20×). (**C**) ISBG granules are integrated and in close contact with the newly formed bone six months post-implantation. Osteocytes and osteoblasts can be detected in the newly formed bone. Soft tissue area is rich with cells, mainly fibroblasts. Note the dark red areas that indicate new bone formation at the ISBG and bone contact. No signs of inflammatory tissue response towards the implanted ISBG. NB, newly formed bone; BM (biomaterial), residual ISBG; ST, soft tissue; black filled triangles: osteocytes; unfilled triangles: osteoblast; arrows: fibroblasts (Movat’s pentachrome staining, magnification 20×).

The viscosity of ISBG allowed easy manipulation during surgery and successful application into the bone defect without any leakage. The healing period was uneventful; there were no infection complications or postoperative pain. Histological examination showed a homogenous trephine biopsy containing residual ISBG surrounded by newly formed bone and soft connective tissue. The residual ISBG had an irregular appearance and was easily detectable by both stainings (Figure 3). Osteoconductive growth started in the peripheral region where the ISBG and pristine bone were in close contact. Newly formed bone contained osteocytes trapped in a mineral tissue, while active osteoblasts were found at the boundary between the newly formed bone and the remaining ISBG, indicating bone remodeling (see Figure 3B,C). The soft tissue area was rich in fibroblasts. There were no signs of inflammatory tissue reaction, implying that the ISBG is biocompatible with the surrounding tissue. Finally, the histomorphometric examination revealed 24.76% mineralized tissue, 12.56% ISBG and 62.68% soft tissue.

**Figure 4:**
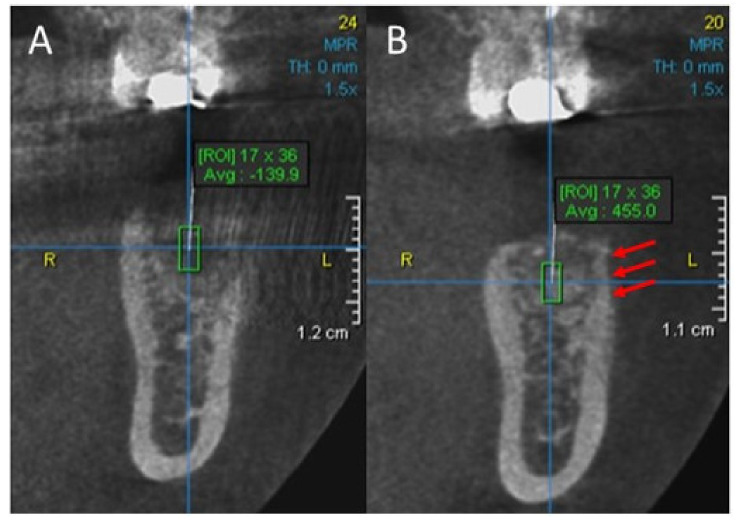
Grey levels in rectangular ROI at the site of augmentation. (**A**) CBCT scan ten days postoperatively. (**B**) Cone beam computed tomography (CBCT) scan 6 months post augmentation with ISBG. Pay attention to buccal bone plate regeneration at the site of augmentation (red arrows).

The tissue densities in grey level are compared in ROI as presented in Figure 4. Average ROI grey level was 138.5 in the first and 454 in the second CBCT scan taken. Standard deviations as measure for noise, showed no differences between images. Furthermore, imaging showed restitutio at integrum of the buccal bone plate (Figure 4B). The average grey level at the site of buccal bone plate regeneration was determined to be 728 using 1 mm × 3.6 mm ROI.

## 4. Discussion

The present clinical case report demonstrated that the use of ISBG generated clinically, histologically and radiologically satisfactory regeneration of buccal fenestration. The ISBG was integrated and in close contact with the newly formed bone six months post-implantation. Osteocytes were trapped in newly formed bone, while active osteoblasts were found at the border between the newly formed bone/residual ISBG and no signs of inflammatory tissue reaction were observed.

Previous study of ISBG showed that it is easy to handle and can serve as an excellent BMP9 carrier in order to demonstrate its in vitro osteoinductive potential [32]. Another in vivo study showed that ISBG was gradually invaded by cells and complex tissue elements [26]. Here it was observed that ISBG can build a barrier-like structure and with that was able to control the soft tissue influx into the implantation bed. Furthermore, the biomaterial-associated multinucleated giant cells response was significantly expressed; which can influence the process of angiogenesis and is in accordance with the GBR concept.

GBR with simultaneous implant placement and ISBG in a combination with pericardium collagen membrane showed complete bone defect regeneration, long-lasting volume stability and soft tissue aesthetics in patients that were monitored for 2–5 years [24]. Even though ISBG showed higher volumetric loss than bovine xenografts in maxillary sinus floor augmentation procedures after three years follow up, there was no significant difference in new bone formation between 6th and 9th month [25,33]. Here the mean percentage of new bone was 15% and 21%, respectively. This is in accordance with our observation that at six months the amount of soft tissue was higher than the new bone formation. However, this can be attributed to the different indications. A complicated defect such as buccal fenestration would most likely require the use of a longer lasting barrier such as pericardium membrane or a non-resorbable membrane. Though, according to Soldatos, in combination defects both membranes give good results as long as the membrane is not exposed during healing, which we have successfully achieved [34].

CBCT imaging was done to compare tissue density during two stages of the implantation process with histological outcome. During the six months between imaging, the tissue density presented as mean grey level increased significantly (Figure 4A,B), which supports histological findings at the augmentation site.

The current case report shows that ISBG is very easy to handle and caused successful healing in the buccal fenestration defect. Still, more studies should be done to observe results reproducibility in larger number of patients.

## 5. Conclusions

Injectable synthetic bone graft has been used for regenerative treatments for many years. However, its efficacy in management of buccal fenestration defect remains incompletely investigated. Therefore, we examined the histological outcome with ISBG in a patient having buccal fenestration. Six months post-implantation we observed good integration of the ISBG into the newly formed bone, without any signs of tissue inflammation response. Moreover, CBCT analysis confirmed buccal bone plate regeneration. Based on the histological and radiological results of this case report, we can conclude that the use of ISBG in the treatment of buccal fenestration leads to successful bone regeneration. However, an additional long-term clinical study with a suitable population is needed to complete the histological and radiological evidence for the efficacy of ISBG in bone regeneration.

## Data Availability

The data presented in this case report are available on request from the corresponding author.
